# Salivary Markers of Oxidative Stress in Patients Undergoing Orthodontic Treatment with Clear Aligners versus Self-Ligating Brackets: A Non-Randomized Clinical Trial

**DOI:** 10.3390/jcm11123531

**Published:** 2022-06-20

**Authors:** Cristina Menéndez López-Mateos, María Luisa Menéndez López-Mateos, Antonio Aguilar-Salvatierra, Gerardo Gómez-Moreno, Javier Carreño Carreño, Hoda Khaldy, Mario Menéndez-Núñez

**Affiliations:** 1 Department of Stomatology IV, Faculty of Odontology, Complutense University, Plaza de Ramón y Cajal s/n, 28040 Madrid, Spain; cmenendezlm@gmail.com; 2 Faculty of Dentistry, University of Granada, Colegio Máximo de Cartuja s/n, 18071 Granada, Spain; menendez@correo.ugr.es (M.L.M.L.-M.); aaguilar@ugr.es (A.A.-S.); ggomez@ugr.es (G.G.-M.); 3 Faculty of Dentistry, European University, 28670 Madrid, Spain; drjaviercarreno@gmail.com; 4 Scientific Instrumentation Center, University of Granada, Paseo Professor Juan Osorio s/n, 18071 Granada, Spain; hkhaldy@ugr.es

**Keywords:** clear aligners, fixed orthodontics, self-ligating brackets, saliva, advanced oxidation protein products (AOPPs), total antioxidant capacity (TAC), myeloperoxidase activity

## Abstract

The aim of this work was to determine advanced the oxidative protein products (AOPPs), total antioxidant capacity (TAC), and myeloperoxidase activity (MPO) in the saliva of patients undergoing orthodontic treatment with clear removable aligners in comparison with another group in treatment with fixed passive self-ligating brackets applying light forces, before treatment, after 30 days, and after 90 days of treatment. This non-randomized clinical trial recruited patients consecutively, all of which were over 18 years of age and due to undergo orthodontic treatment. They were divided into two groups according to treatment type: Group A, 48 patients treated with clear aligners (Invisalign^®^); and Group B, 19 patients treated with Damon System^®^ 0.22″ self-ligating brackets applying light forces. Saliva samples were collected by a single clinician following the same protocol and underwent three analyses—AOPPs, TAC, and MPO levels–at baseline before placing the apparatus, after 30 days, and after 90 days treatment. Orthodontic treatment, whether with clear aligners or fixed self-ligating brackets and light forces, increased AOPPs after the first 30 days of treatment. During the initial phases of orthodontic treatment, neither clear aligners nor fixed self-ligating brackets applying light forces showed changes in TAC and MPO. Orthodontic treatment with both clear aligners and fixed apparatus self-ligating brackets applying light forces increases oxidative stress (AOPPs) after the first 30 days of treatment. There are no differences in AOPP levels between treatment with clear aligners and self-ligating brackets during the first 90 days of treatment. The antioxidative capacity of saliva during the initial phases of orthodontic treatment, whether with self-ligating brackets or clear aligners, does not undergo significant changes. With either orthodontic technique, the patients’ salivary antioxidant capacity is similar.

## 1. Introduction

Saliva is an essential feature of the oral cavity and to some degree salivary markers reflect the mouth’s condition and health [1]. In recent years, saliva has become a widely-used diagnostic means in clinical research. Its availability, easy collection, and the fact that samples may be taken repeatedly and non-invasively makes saliva an ideal means of screening, diagnosing, monitoring, or conducting research into a range of diseases [2]. Saliva conserves oral health, participates in the oral defense of the organism, and maintains the equilibrium of the oral environment [3].

Oxidative stress is defined as the imbalance between the production of free radicals and the organism’s capacity to arrest or minimize their damaging effects through neutralization by anti-oxidants [2]. Physiologically, a dynamic equilibrium exists between the set of free radicals that have the capacity to produce oxidative damage (reactive oxygen species—ROS) and antioxidative defense capacity. The defense mechanisms of normal cells destroy most of these ROS and free radicals [4]. Oxidative stress occurs when the intracellular concentrations of reactive oxygen species exceed physiological values. The cytotoxic effect of free radicals on cells is harmful and will lead to cell damage by affecting the peroxidation of double bond fatty acids, proteins, and DNA [5].

In orthodontic treatment, two different situations coexist that can trigger oxidative stress: on the one hand, the apparatus itself and on the other, the biomechanics of dental movement [1]. The use of an orthodontic apparatus in the treatment of malocclusions creates a complex environment in the oral cavity. This often provokes an inflammatory response around the teeth undergoing displacement [2]. During treatment, various inflammatory mediators (cytokines) are released, causing the aseptic inflammation of the periodontal ligament when mechanical forces are applied to the teeth. This brings about a chain of reactions in the periodontal ligament involved in tissue remodeling and dental movement [6]. One of the biological reactions to orthodontic treatment and subsequent inflammation in the oral cavity is the oxidative stress associated with a series of attenuated pro-inflammatory factors [2]. As there is solid evidence to indicate that periodontal inflammation is one of the main sources of ROS in the oral cavity, it is plausible to suppose that aseptic inflammation could be associated with the damage caused by oxidative stress [5].

Most orthodontic apparatus is made from metallic, ceramic, or plastic materials, which can release metals or other elements due to the corrosion of the apparatus. This can increase ROS levels through different reactions with those free radicals that are catalyzed by metals [5]. Orthodontic biomaterials exert an influence on the oral environment and undergo complex reactions with different components. Their impact on various salivary parameters has not been clearly elucidated, despite recent innovations in orthodontic biomaterials and the nature of tissue–biomaterial interactions. To date, research has not determined the specific correlation between placing an orthodontic apparatus in the oral cavity and the precise biological and clinical outcomes of this action [3].

Biomechanical dental movement is another factor that influences periodontal inflammation. Traditionally, orthodontic forces have been classed as ‘light’ or ‘heavy’ and it has been assumed that light forces are softer and therefore more physiological, as well as offering other advantages such as the optimal treatment time, friction, and esthetics [7]. At present, the most widely used light force orthodontic techniques are clear aligners made from plastic, or passive self-ligating brackets combined with superelastic archwires.

Recently, it has been postulated that individual markers could validate the presence or prognosis of disease; nevertheless, analyzing a set of markers will be more useful and more appropriate for determining advanced oxidative protein products (AOPPs) and the total antioxidant capacity (TAC) [1]. The hypothesis of the present study was that the comparison of the orthodontic techniques would exhibit significant differences in the behavior of the variables analyzed. The aim of this study was to determine the salivary markers of oxidative stress—advanced oxidative protein products (AOPPs), total antioxidant capacity (TAC), and myeloperoxidase activity (MPO)—in patients undergoing orthodontic treatment with removeable clear aligners compared with another group treated with fixed passive self-ligating brackets applying light forces, before the start of treatment, after 30 days and after 90 days treatment.

## 2. Materials and Methods

### 2.1. Ethics Committee

The study design followed ethical guidelines for research involving human subjects established in the Declaration of Helsinki (revised version, 2002). All patients were provided with information about the nature of the trial and gave their informed consent to participate by signing a form. This study was approved by the University of Granada Committee for Research Ethics (Registration Number 549/CEIH/2018).

### 2.2. Study Design and Subjects

This non-randomized, clinical trial recruited patients consecutively, all aged over 18 years and seeking orthodontic treatment at a private clinic in Granada (Spain). Purposive sampling was carried out due to the difficulty of recruiting subjects given that the study aimed to monitor the behavior of three variables and their possible interaction with different types of orthodontic treatment. This study was conducted between May 2018 and June 2021. Patients were divided into two groups according to treatment type: Group A included 48 patients treated with clear aligners (Invisalign^®^); and Group B consisted of 19 patients treated with Damon System^®^ 0.22″ self-ligating brackets, applying light forces. All patients attended all follow-up sessions; there were no drop-outs. All patients were compliant with the study protocol throughout. They were treated with the same standard procedures, by the same clinician, and at the same clinic. The treated malocclusions had similar characteristics: slight or moderate overcrowding treated without dental extractions. Detailed clinical notes were prepared for each patient after general extraoral and intraoral examination. To complete diagnosis, three-dimensional digital study models were produced with an iTero^®^ Element Intraoral Scanner (Align Technology, Inc., San Jose, CA, USA); intraoral and extraoral photographs, orthopantomographs and lateral teleradiography images of the cranium were also taken.

Exclusion criteria were: patients presenting active periodontal disease at the start of the study, patients who had received fixed orthodontic treatment within the preceding 24 months, patients who had been taking antibiotics or anti-inflammatories during the week preceding the initial examination and saliva collection, or any patients who did not provide their informed consent to take part.

### 2.3. Treatment with Clear Aligners

The Invisalign system (Align Technology, San Jose, CA, USA) is a new generation of clear, semi-elastic, multi-layer polyurethane removable aligners. The system is made of thin transparent plastic, composed of polymer chains of organic units joined by urethane links. Each aligner is digitally designed for placement on the oral, lingual, palatine, and occlusal surfaces of the teeth [8]; it is currently fabricated from a material known as SmartTrack™. The teeth are gradually moved towards their final positions, planned in advance by means of dedicated software (ClinCheck^®^, Align Technology, Inc., San Jose, CA, USA), which specifies both the treatment sequence and the type and magnitude of movement of each tooth. Patients are given an information leaflet specifying the date when aligners should be changed and how long they must be worn (22 h per day).

On the first day of treatment, aligner 1 was delivered and placed without attachments on the teeth. Each patient returned to the clinic 7 days later when aligner 2 was delivered, this time with attachments on the teeth, also supplying the next two pairs of aligners. Patients were asked to return for the second saliva collection 30 days after the start of treatment. At this point, subsequent aligners were delivered to the patient to be changed weekly, carrying out regular check-ups. Patients were asked to return 90 days after the start of treatment for the third saliva sample collection.

### 2.4. Treatment with Fixed Apparatus

Group B was treated with Damen Q passive self-ligating brackets (Ormco Corporation, Orange, CA, USA) combined with Damon Optimal Force Copper Ni-Ti^®^ 0.014″ archwires (Ormco Corporation, California, USA) as the first wire in the treatment sequence. Patients returned 30 days later for the second saliva sample collection and to receive the second archwire: Copper Ni-Ti^®^ 0.018″. At the end of the second month, Copper Ni-Ti^®^ 0.014″ × 0.025″ archwires were placed (Ormco Corporation, Orange, CA, USA). After 90 days, the third saliva samples were collected and the archwire sequence continued.

### 2.5. Saliva Sample Collection

All saliva samples were collected by the same clinician following a validated, previously published protocol [9] at baseline before starting treatment, and then at 30 and 90 days after the start of treatment before placing the next apparatus in the sequence. Stimulated saliva flow was obtained between 08.30 and 09.00 in the morning. Patients chewed a paraffin tablet for 5 min (at least 1 h since food and/or drink had been taken). The saliva secreted during the first 2 min was eliminated. After that, saliva was collected in a plastic container for a further 5 min. Saliva was collected before any type of intra-oral intervention was carried out (examination, tooth-brushing, etc.). The saliva samples were frozen at −80 °C and later centrifuged at 3000 rpm for 20 min, and then placed in Eppendorf tubes and frozen again at −80 °C.

### 2.6. Advanced Oxidative Protein Product (AOPPs) Measurement

AOPPs in saliva were determined by means of a spectrophotometric technique as described by Hanasand [10] and Sampson [11]. The results were analyzed with the Ensight^®^ multimode plate reader (PerkinElmer, Waltham, MA, USA) and MyAssays^®^ Desktop PRO data processing (MyAssays Ltd., Brighton, UK). AOPP concentration was expressed as equivalent μM of chloramine-T [12].

### 2.7. Total Antioxidant Capacity (TAC) Measurement

A colorimetric test was used to measure TAC based on the capacity of the antioxidants present in saliva to reduce a preformed radical cation. The principle behind this test is that at an acidic pH in the presence of an adequate oxidant solution (FeCl_3_), chromogenic DMPD (*N,N*-Dimethyl-1,4-Phenylenediamine Sulfate) forms a stable, colored radical cation. When the solution is mixed with saliva, the antioxidant molecules (AOH) present transfer a hydrogen atom from the chromogenic radical’s cation, producing a discoloration of the solution in proportion to the antioxidants present in the sample [13]. The chromogenic radical cation is measured at a wavelength of 550 nm. TROLOX (soluble vitamin E analogue) was used as a standard model to calculate the sample’s total antioxidative activity. The results were analyzed with the Ensight^®^ multimode plate reader and MyAssays^®^ Desktop PRO data processing. The results were expressed as total the antioxidant activity relative to TROLOX (µM).

### 2.8. Myeloperoxidase Activity (MPO)

MPO activity was determined by means of the method proposed by Sakamoto et al. with a slight variation: substituting the chromogen 3,3′-diaminobenzidine (DAB) for another (o-Dianisidine). The results were expressed as mUl/mL [14].

### 2.9. Statistical Analysis

The samples’ descriptive statistics were calculated with software packages IBM SPSS Statistics for Windows Version 22.0. (IBM Corp., Armonk, NY, USA) and R (version 4.0 Vienna, Austria. URL https://www.R-project.org/, accessed on 29 May 2022). The variables compared between the two groups at baseline, 30 days, and 90 days were: TAC, AOPP, and MPO. Repeated-measurement ANOVA was performed to make the intra-group and inter-group comparisons with a significance level of *p* < 0.05 (alpha error = 0.05).

## 3. Results

**Figure 1:**
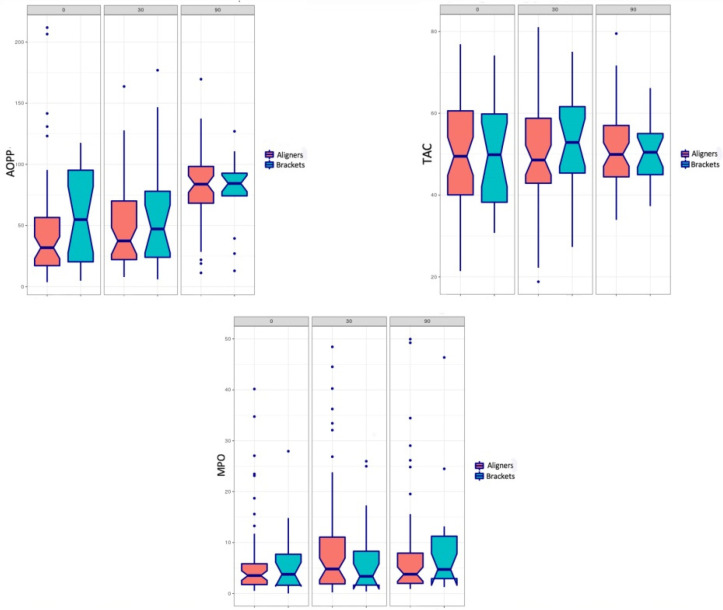
Box plots show median values for variables Advanced Oxidative Protein Product (AOPP), Total Antioxidant Capacity (TAC), and Myeloperoxidase Activity (MPO) during the 90-day study period (T-0, T-30, and T-90 days) for the two orthodontic techniques.

Table 1 shows the sample parameters in relation to the orthodontic treatment groups (Groups A and B). At baseline, mean AOPP values were similar in both groups. The box plot in Figure 1 shows the median values of the variables measured during the study period (0, 30 and 90 days). Table 2 compares the mean AOPP values between the two treatment groups at the three evaluation times (0, 30, and 90 days). Statistically significant differences were found for patients treated with aligners between the baseline and 90 days, and between 30 days and 90 days. Comparing the two orthodontic techniques, no significant differences were found between groups during the 90-day study period. Table 3 and Table 4 provide variance analysis values for the variables TAC and MPO. No statistically significant differences were found for either variable at any of the study times (whether intergroup or intra-group).

## 4. Discussion

One of the negative properties of oxidative stress is the damage it causes to proteins. This is because it can provoke a loss of the catalytic activity of enzymes, damage the integrity of structural proteins, and upset the regulation of metabolic pathways. Unlike nucleic acids, protein repair systems only act on methionine residue, and so oxidated proteins must be hydrolyzed in order to avoid their diffusion in the metabolic network and interaction with other proteins. Reactive oxygen species (ROS) can provoke the oxidation of tyrosine residues. As a consequence, tyrosine dimers form, which provoke the aggregation, crosslinking, and fragmentation of proteins. The set of composites formed by these processes are known as advanced oxidative protein products (AOPPs). As well as being indicators of oxidative stress, AOPPS also reflect myeloperoxidase-dependent chlorination activity, a part of the inflammatory response [10]. The orthodontic techniques compared in the present trial—clear aligners and self-ligating brackets—both applied light forces. Most comparable studies have investigated traditional orthodontic treatments but very few have evaluated self-ligating brackets. As far as we are aware, none have investigated clear aligners. Statistically significant differences were observed in the aligner group between the baseline and 90-day measurements, as well as between the 30-day and 90-day study measurements. Buczko et al. obtained a marked increase in salivary oxidative stress markers 1 week after placing the orthodontic apparatus [1].

During the first 30 days of treatment with clear aligners, very light forces were applied to activate and prepare the periodontal ligament for subsequent dental movement. As Nucera et al. have affirmed, an essential feature of clear aligners is the presence of composite attachments, which determine the amount and quality of dental movement. As such, clear aligners offer advantages in planning and sequencing dental movements [15]. Unlike conventional orthodontic techniques, the sequence of dental movements, the need for anchorage, as well as the type and magnitude of movement are all planned in advance. The technique is able to modulate a range of dental movements. In principle, the magnitude of movement induced by aligners falls within a range of 0.25 mm and 2° rotation movement. These conditions explain why, during the first 30 days, AOPP values remained practically unchanged in the aligner group.

The patient group treated with self-ligating low-friction brackets showed similar behavior to the aligner group. This may be explained by the light forces applied in low-friction techniques. The similar values obtained during the first 30 days suggest that light force techniques do not have much influence on oxidative stress during the first month of treatment. Other trials have made the same claim, arguing that light forces are a possible cause of increase, although not the only one [16]. The esthetics, comfort, and ease of adaptation provided by low force techniques are of benefit to the patient. It is also very likely that such techniques do not generate oxidative stress during the first month of treatment. In orthodontic treatment, it is important to minimize friction. This can be achieved by opting for self-ligating brackets as in the present study (eliminating elastic ligatures), or by means of archwires’ surface treatment as affirmed in the review published by Baçela et al. [17].

In vivo studies that set out to evaluate the salivary markers of oxidative stress or in the gingival crevicular fluid of patients undergoing orthodontic treatment have obtained varying results [7,18]. On the one hand, Buczko et al. found that orthodontic treatment did modify the oxidative–antioxidative balance in saliva [1]. On the other, Atung Ozcan et al. concluded that oxidative stress biomarker levels did not change after 1 or 6 months’ treatment [18]. The present study had a 90-day follow-up, following the protocols of various published works. Atuğ Özcan et al. [18] showed that most oxidative stress occurs during the first phases of orthodontic treatment due to the inflammatory reaction triggered as dental movement begins. Thereafter, the organism adapts, and the neutralization of free radicals takes place.

In the present work, no significant differences in the AOPP levels were found between the group treated with clear aligners and the group treated with self-ligating brackets—a result which was quite unexpected. The self-ligating bracket system with superelastic archwires has a low load-deflection quotient. This makes it possible to apply an optimal force that does not require repair time and can therefore act continuously. It has been suggested that teeth moved by aligners do not undergo the typical phases of orthodontic movement [19], as described by Krishnan and Davidovitch [20]. This is because the forces applied by aligners are intermittent.

Nevertheless, it would appear that the periodontium perceives light continuous orthodontic forces as intermittent [21]. At the same time, intermittent forces would appear to produce dental movement with less cell damage in the periodontium [22]. Therefore, it is likely that it is orthodontic treatment in general that is responsible for these results rather than the specific technique [23]. In the present study, neither clear aligners nor fixed self-ligating brackets applying light forces showed any changes in TAC and MPO, which perhaps may be explained by the fact that both treatments applied light forces.

The present work did not identify statistically significant differences between the study times during treatment. On the basis of these findings, it may be affirmed that orthodontic treatment with self-ligating brackets and light forces does not have much influence on oxidative stress in the oral cavity during the first 90 days of treatment [1,23].

The present work suffered from some limitations, namely the small sample size and sample distribution (mainly consisting of young adults). For this reason, the findings may not be extrapolated to other age groups such as older adults or children. Patients who fulfilled the inclusion criteria were recruited consecutively. However, current local demand for treatment with clear aligners is much higher than for fixed aligners, which explains the difference in the sizes of the two groups. Nevertheless, the authors do not believe that this influenced the final results. Another fundamental limitation was the difficulty of recruiting patients and obtaining informed consent to participate, as many were seeking mainly esthetic improvements and often preferred not to take part. Moreover, many patients were simply unable to come to the clinic early in the morning. This problem, the cause of the small sample size, was the study’s weakest point. The same difficulty is reflected in a study by Pantea et al., who set out to determine the salivary oxidative stress parameters in relation to a resin interim material used in dentistry [24]. Nevertheless, our sample size was larger. In addition, storing saliva samples at −80 °C is difficult and not always possible. Possible fluctuations in the biochemical values and role chance were managed as much as possible by collecting the samples at the same time for all patients (between 08.30 and 09.00 in the morning) and always by the same clinician. However, it is clear that the technical limitations of this clinical trial and the variability among the patient sample made it practically impossible to control all the variables.

Nevertheless, as far as we are aware, this is the first study of this type realized with clear aligners and self-ligating brackets, a fact which will make it of interest to clinicians.

## 5. Conclusions

Orthodontic treatment with both clear aligners and a fixed apparatus self-ligating brackets applying light forces increases oxidative stress (AOPPs) after the first 30 days of treatment. There are no differences in AOPP levels between the treatments with clear aligners and with self-ligating brackets during the first 90 days of treatment. The antioxidative capacity of saliva during the initial phases of orthodontic treatment, whether with self-ligating brackets or clear aligners, does not undergo significant changes. With either orthodontic technique, the patients’ salivary antioxidant capacity is similar.

## Figures and Tables

**Table 1:** Treatment group characteristics and total salivary protein concentration at baseline.

KERRYPNX	Group A Aligners *(n* = 48)	Group B Self-Ligating Brackets (*n* = 19)
**Age (SD)**	32.2 (9.8)	29.3 (9.4)
**Sex**		
Men (%)	7 (14.6%)	4 (21.1%)
Women (%)	41 (85.4%)	15 (78.9%)
**Smoking**		
Yes (%)	11 (22.9%)	7 (36.8%)
No (%)	37 (77.1%)	12 (63.2%)
**Total salivary protein concentration at baseline (SD)**	0.5 (0.3) mg/mL	0.5 (0.1) mg/mL

**Table 2:** Mean AOPP values (SD) (μM) for the two orthodontic techniques at T 0, T 30, and T 90. * *p* < 0.05.

	T 0	T 30	T 90	*p*-Value
Aligners	47.1 (46.9)	50.1 (38.3)	79.5 (33.6)	T0-T30: 0.9 T30-T90: 0.001 * T0-T90: 0.000 *
Self-ligating brackets	57.8 (39.5)	58.6 (46.8)	80.2 (27.7)	T0-T30: 0.9 T30-T90: 0.2 T0-T90: 0.1
*p*-value	0.3	0.4	0.9	

**Table 3:** Mean MPO values (SD) (mUl/mL) for the two techniques during the 90-day study period.

	T 0	T 30	T 90	*p*-Value
Aligners	8.4 (13.7)	13.9 (23.1)	12.7 (19.2)	T0-T30: 0.3 T30-T90: 0.9 T0-T90: 0.5
Self-ligating brackets	14.3 (25.5)	9 (12.7)	12 (17.4)	T0-T30: 0.6 T30-T90: 0.8 T0-T90: 0.9
*p*-value	0.2	0.3	0.9	

**Table 4:** Mean TAC values (SD) (μM) for the two techniques during the 90-day study period.

	T 0	T 30	T 90	*p*-Value
Aligners	50 (13.7)	49.8 (13.5)	51.6 (10.4)	T0-T30: 0.9 T30-T90: 0.7 T0-T90: 0.9
Self-ligating brackets	49.1 (13.7)	53.5 (12.5)	49.8 (7.6)	T0-T30: 0.4 T30-T90: 0.5 T0-T90: 0.521
*p*-value	0.8	0.3	0.4	

## Data Availability

Not applicable.
